# Schistosomiasis infection in pre-school aged children in Uganda: a qualitative descriptive study to identify routes of exposure

**DOI:** 10.1186/s12879-019-3803-z

**Published:** 2019-02-14

**Authors:** Simon Peter Sebina Kibira, John C. Ssempebwa, Ronald Ssenyonga, Scott Radloff, Fredrick Edward Makumbi

**Affiliations:** 1 Department of Community Health and Behavioural Sciences, School of Public Health, College of Health Sciences, Makerere University, Kampala, Uganda; 2 Department of Disease Control and Environmental Health, School of Public Health, College of Health Sciences, Makerere University, Kampala, Uganda; 3 Department of Epidemiology and Biostatistics, School of Public Health, College of Health Sciences, Makerere University, Kampala, Uganda; 4 Department of Population, Family and Reproductive Health, Johns Hopkins Bloomberg School of Public Health, Baltimore, USA; 5 Department of Epidemiology and Biostatistics, School of Public Health, College of Health Sciences, Makerere University, Kampala, Uganda

**Keywords:** Schistosomiasis, Bilharzia, Uganda, Qualitative, Children, Neglected tropical diseases, Water, Sanitation

## Abstract

**Background:**

Prevalence of schistosomiasis is high among children under five years in Uganda. Schistosomiasis control efforts over time have included periodic mass treatments in endemic areas for adults and school going children aged 5 years and above. This study explores behaviour practices of children age 2–4 years that increase the risk of schistosomiasis infection in this age group.

**Methods:**

A qualitative descriptive study was conducted using in-depth interviews with 30 caregivers of children aged 2–4 years who tested positive for schistosomiasis in a national prevalence survey in 2017. Observations were done at water bodies where young children go with caretakers or other older children. The study was conducted in three Ugandan sub-regions of West Nile and East-central, and South-western with high, and low prevalence of schistosomiasis, respectively. Data were thematically analysed. Anonymised supporting photos from observations are also presented.

**Results:**

Knowledge about schistosomiasis transmission was poor among caregivers, who concurrently had mixed right and wrong information. Reported avenues for contracting schistosomiasis included both correct: contact activities with infested water, and incorrect modes: contact with dirty water, sharing bathrooms, witchcraft, polluted air and contaminated food. The children in this study could have contracted schistosomiasis through the contact with infested water during activities such as bathing and playing, while their caregivers washed clothes, collected snail shells for poultry feeds, fetched water at the water bodies. These activities were reported by caregivers and observed in all study areas. Evidence of open defecation and urination in and near water bodies by adults and children was also observed.

**Conclusions:**

Pre-school children age 2–4 years are at a high risk of exposure to schistosomiasis while caretakers conduct activities in infested water bodies. There is need for prevention interventions to target children in their early stages of life to control schistosomiasis in this vulnerable population.

## Background

Schistosomiasis is an acute and chronic disease caused by parasitic worms. It is one of the Neglected Tropical Diseases (NTDs) that were listed for control by the year 2020 at the 2012 London declaration on NTDs [1] and by the World Health Organisation (WHO) [2]. It remains a huge public health challenge in many parts of the world including Uganda. The schistosomiasis transmission cycle starts when human urine and faeces containing parasite eggs are deposited in fresh water environments and hatched larvae infect the snail hosts. The parasites multiply in snails, releasing another larval stage into water that infects humans [3]. Infection is transmitted when people are exposed to infested water bodies throughout routine agricultural, domestic, occupational, and recreational activities. The larvae evolve into male and female worms inside the human body and these coexist in the blood vessels for years. Female worms release thousands of eggs that are evacuated in human excreta. The cycle is perpetuated when infected people urinate or defecate in freshwater sources [3]. The lack of proper sanitation and hygiene facilities increases the risk of contaminating the water bodies. School-going children and older preschool- age children are exposed to the parasites through swimming and bathing in infested water, and other water activities [4, 5]. Studies have showed that infants and younger preschool-age children can be exposed by their mothers and caregivers who take them to water sources or use infested water to bathe them. Caregivers have admitted to these exposures in studies in Nigeria [5, 6]. The disease is estimated to affect more than 240 million individuals worldwide and millions of people including children live at risk.

The WHO guided schistosomiasis control efforts are focused on reducing the disease through periodic largescale mass treatments with praziquantel [4]. However, by the year 2016, the WHO estimated that only 36% of the people requiring preventive treatment for schistosomiasis were reached [4]. This leaves a big gap in the control efforts. Other strategies to sustain control and advance towards elimination include behavioural based interventions such health education for behavioural change to prevent water contamination and contact with infested water; interventions to provide safe water, sanitation and hygiene; environmental management and; snail control [3].

For preschool-aged children, there are no approved recommendations appropriate for the mass treatment, although there have been suggestions to use the single dose of praziquantel of 40 mg/kg, recommended by the WHO for *S. mansoni* infections in school-aged children [7]. Paediatric formulations of praziquantel are still under clinical development to enable inclusion of preschool-aged children in preventive chemotherapy programmes [8]. Several gaps and challenges in treatment have been documented [9–12], which include the absence of easy to use, child size, medicines [6]. In the current state, the WHO recommends that in schistosomiasis endemic areas, treatment for preschool-age children should be included in the regular health services, and ongoing public-health interventions such as child health days and expanded programme on immunization [8]. There are challenges to this recommendation in Uganda; foremost is that Praziquantel is not readily available at health facilities to facilitate such efforts and, there are no diagnostic kits at health facilities. In the absence of an appropriate paediatric formulation, the WHO recommends that broken or crushed tablets can be used for administration of praziquantel [8]. However, these are not feasible in mass treatment campaigns given the complexities involved in the preparation.

The challenges above render efforts to treat children hardly possible and thus puts them at increased risk of developing serious consequences including death. Schistosomiasis eggs that are not passed out get trapped in body tissues resulting in immune reactions which lead to progressive damage to the body organs [4, 13]. Therefore the delayed treatment of the under-five children will affect their wellbeing [14] and growth [13] at this vital stage of development. Yet, studies in Uganda [11, 15] and elsewhere [6, 16, 17] showed that preschool-age children have higher infection levels than the adults. In Uganda, the first nationally representative population-based schistosomiasis sample survey conducted in 2016 showed a prevalence of 22%. The prevalence significantly varied by age, most common among 2 to 4-year old children (31%), followed by 13 to 19 years (28%) and lowest in the 20 and above years (19%) [15]. In Uganda mass preventive chemotherapy mainly focuses on primary school going age children, following available guidelines.

It is vital to have a better understanding of the exposure to schistosomiasis among under-five children to inform the design of effective context specific interventions for the prevention and treatment of this NTD. It is also crucial to have a clearer understanding of how early the infection occurs and the social-cultural factors, as well as the role of the caregivers in exposing children to Schistosomiasis [6]. This study aimed to explore the behavioural practices related to water, sanitation and hygiene among children under-five, who tested positive for schistosomiasis and their caregivers in the national prevalence survey of 2017. The study team also observed activities around waterbodies in communities where these children had been surveyed.

## Methods

### Design and scope

This sub-study was part of the larger study that used a sequential explanatory mixed methods approach. This sub-study was a qualitative descriptive design [18, 19] that sought to explore the practices that potentially exposed children aged 2–4 years, who tested positive with schistosomiasis in the 2017-round two schistosomiasis national survey, conducted in all the sub-regions. The qualitative description [18] was conducted in three purposely selected sub-regions of Uganda, with high (west Nile and East Central) and low (South Western) prevalence of schistosomiasis. The prevalence was based on the 2016-round one PMA2020 schistosomiasis national survey [15]. Eight enumeration areas/villages were selected from those where the national survey was conducted (three in both East Central and West Nile regions, and two in South Western region).

### Data collection and participants

In the eight enumeration areas, the research team purposely selected households with schistosomiasis infected children aged 2–4 years, based on the Point-of-Care Circulating Cathodic Antigen (POC CCA, Rapid Medical Diagnostics, Pretoria, South Africa) rapid test results in round two of the survey [20]. Participants interviewed were the caregivers of the infected children. Ten caregivers of the infected children in each enumeration area were selected, resulting in 30 participants (Table 1). In-depth interviews of the caregivers were conducted by three experienced research assistants to establish the behaviours surrounding the potential exposures to cercaria-contaminated water. To triangulate the findings from the in-depth interviews, non-participant observations for practices around the main water bodies in each enumeration area, as well as the sanitation and hygiene status around the community, were conducted. A total of five observations were conducted. Photographs were also taken to provide evidence of the observed practices.

**Table 1:** Distribution of the caregivers of schistosomiasis-infected children, and the water body observations, by location

Regions and district	Villages/EAs covered	Number of in-depth interviews with caregivers	Number of observed water bodies
South west (Mbarara)	2	10	1
East central (Jinja and Bugiri)	3	10	2
Northern (Arua)	3	10	2
Total	8	30	5

### Data management and analysis

All audio recorded local language interviews were transcribed and translated into English. The transcripts were proof-read before importing them into atlas.ti7, a qualitative data management software. Data coding and analysis were conducted subsequently. The first author together with a team of three interviewers developed an initial codebook using a sample of transcripts. The developed codebook was later applied to the entire atlas project by two coders with any emerging codes added in the process. The query reports and code-document tables were used to establish similarities in patterns and the magnitude of categories respectively. Thematic analysis was used, and results are presented using themes with typical quotations from the caregivers’ interviews to support the evidence. Data from observations are presented thematically with photographs as supporting evidence. This manuscript was prepared following the Qualitative Research Review guidelines- RATs criteria.

## Results

A total of 39 under-five children who tested positive for schistosomiasis were linked to their 30 caregivers during the survey (Table 2). Most of the schistosomiasis positive under-five children were females (21) and aged four years (19), while nearly all caregivers were female (28), with a median age of 28.5 years. The majority of the caregiver had primary level education (17) and were small-scale farmers (16).

**Table 2:** Characteristics of the children positive with schistosomiasis and their caregivers

Characteristic	Number
*Sex of caregiver*	
Female	28
Male	2
*Age of caregiver*	
18–24	9
25–34	10
35+	11
*Education of caregiver*	
None	4
Primary	17
Secondary+	9
*Main occupation of the caregiver*	
Farming	17
Petty trading	7
Housewife	2
Casual Labour	2
Formal employment	2
*Age of the children*	
2 years	17
3 years	3
4 years	19
*Sex of children*	
Female	21
Male	18
Total children in the study	39

The findings are organised into the following themes: i) Caregivers’ general knowledge about schistosomiasis and sources of information; ii) Caregivers’ perceptions about positive test results and expectations; iii) Perceptions of how children were infected and the stage of infection. The themes from the observations are categorised according to the various risky activities that expose children to infested water.

### Caregivers’ general knowledge about schistosomiasis and sources of information

One third (20) of the caregivers in this study had information about how schistosomiasis is transmitted from person to person. However, there was a mix of right and wrong information from the same caregivers. Misconceptions were rife even among caregivers that also had the right information. Contact with infested water, dirty water, sharing bathrooms, witchcraft, polluted air and contaminated food were some of the reported avenues for infection. The caregivers’ report sources of information were the health workers at the health facilities and community health workers, radio, community members and research assistants for the survey.

Majority of the caregivers (18/30) mentioned that contaminated river water when infected people urinate or defecate in the water was the main source of infection. They reported stepping in the infested water bodies as responsible for contracting the parasites. However, caregivers who reported dirty water (21/30) had variants of what comprised “dirty” in the context of schistosomiasis transmission. The descriptions varied from muddy water, water that has been used for hand washing in one bowel by several people, and surface water collecting after rainfall that is muddy or containing garbage.

A few (4/30) caregivers wrongly believed that sharing a bathroom with an infected person transmits schistosomiasis. They reported that if a person who has uses urinates in the bathroom while bathing, they, they could spread the disease to other users. Other rare misconceptions included schistosomiasis being air borne (1/30). One person also reported that eating vegetables that are not well cleaned post-harvest and well cooked is a source of parasites.

*The ways in which schistosomiasis is got are through dirty water, use of dirty utensils, and dirty food eaten.* **P36_west Nile, 23 years**

*I knew that using dirty [unclean] water from a well causes schistosomiasis, urinating in the same area [with infected people] or using one urinal causes schistosomiasis. But generally, it is poor hygiene.* **P9_east central, 25 years**

Two caregivers believed that they could be infected through witchcraft, while one believed that schistosomiasis is acquired after having persistent malaria, *‘because a child who always has malaria, urinates yellow urine’*. This caregiver further believed that if a sick child is not given appropriate malaria treatment, the eyes turn yellow and the stomach swells and hardens.

When asked about the common symptoms of schistosomiasis, most caregivers reported swollen stomach, losing weight, having pale skin and general body weakness.

*“It is a kind of disease that makes the stomach swell”* **P15_south western, 43 years**

A few caregivers (5/30) confessed having no knowledge about schistosomiasis until they talked to the research assistants in the survey. They took more interest in the diseases after testing them and/or their children and receiving a positive test result(s).

*“No, I did not know anything, but I got to know from you (Research assistants)”* **P4, _east central, 65 years**

*“I did not know about schistosomiasis, but I also tested positive. I do not know how I got it”* **P37_west Nile, 38 years**

The reported sources of information about schistosomiasis were mainly the health workers at facilities and community health workers the radio, community members, schools, and the survey research assistants. Half of the caregivers had received information from health workers while the radio was the second most mentioned source (14/30). A few caregivers (8/30) reported schools and the fellow community members as a good source of information. None of the caregivers had seen or found any message on schistosomiasis from printed messages pinned up in any place in the community or in the newspapers. Several (8/30) of the caregivers had not taken keen interest in the disease until the community health workers moved around the community with the research assistants conducting the survey part of this study. Some of the prevention information they reported to have received included boiling water or letting it settle for some hours in the sun before using it for any activity, avoiding contact with swamps, channels or river water.

*“I got this information through the media on radio, a station called radio west…. We were sensitized on the causes of schistosomiasis and that it has affected many people. They also educated us how one is infected with schistosomiasis like using un-boiled river/well water, and stepping in contaminated water”* **P16_south western, 23 years**

### Perception about test results and expectations

The positive test results of the children were received with mixed feelings. All caregivers except two, were visibly worried that their children were infected. Their most worry was because their children could not be treated like other people in the survey. This also caused disappointment among caregivers. In cases where the children had been ill or had symptoms of the disease, this created more worry about the possible consequences of remaining untreated; now with evidence after the diagnosis. There were also concerns among caregivers whose children looked healthy and yet had received a positive diagnosis.

*“I felt that was bad [not being treated] because she was positive. But I wanted the medicine you [research team] gave others, so that the schistosomiasis clears from her body”* **P29_west Nile, 34 years**

The concern about non-availability of drugs from the survey for their children was widely expressed. Caregivers were left pondering about what to do. Even though they were referred to health facilities, they said there were no drugs available for their children under five years. A few had tried with urgency after the testing but were disappointed at the health facilities.

*“Now if they have not yet brought the medicine, what else can you do? I was told to go to the health facility to get help, but when I went there, they did not have the medicine”* **P14_south western, 28 years**

There were two caregivers in the endemic area (East central district of Jinja) who did not express any surprise or worry about the positive test results. The tests only served as a confirmation of their expectation. This was because their children were exposed to the same conditions as the adults that were receiving treatment from mass drug administration by the Ministry of Health’s Vector Control Division.

*Anyway, I did not take it to be that urgent because it is not a disease that requires immediate attention or needing money immediately. I took it as a ‘by the way’ when I was told she was positive. It is you who is reminding me now.* **P9_east central, 25 years**

Others in the same area were worried but not surprised as well. They had known these risk factors from the radio campaigns that were running at the time.

*“I felt so bad though not so surprised at all because I go with them to the swamp to cultivate. They step in water, we take water from the well because we have no other option, we have no borehole, no tap.”* **P1_east central, 51 years**

A few (3/30) caregivers were instead grateful for the diagnosis, even in the absence of treatment for the children in the survey. But this diagnosis opened their minds about the infection.

Due to the health promotion messages running on the radio stations in the endemic districts, caregivers expressed further worry than they had before the campaigns. They reported that the campaign informed them that schistosomiasis, which they did not take as serious before, slows down children’s growth rates and could kill.

*The way they sensitise on radio, I get to think that it is not easy to treat and can also require a lot of money to heal, many children could die.* **P2_east central, 35 years**

### Potential perceived sources of infection and stage at infection for the children

Caregivers were also asked if they had information on how their children could have been infected with schistosomiasis, and the possible time/stage in their life when they think this had happened. Reported activities included playing in the water while adults washed, exposure at the workplaces of caregivers, and fetching water for domestic use. Some had misconceptions while a few had no idea how the children were infected.

According to the caregivers, their children were engaged in washing clothes at the river banks with them or with their older siblings. Swimming among them was not very common because they were very young, but they played in the shallow ends of the water bodies while their older siblings swam. Caregivers reported that it was hard to control children, especially because when they normally went out to recreate, they were at a time not in the company of adults.

Some caregivers were aware that the kind of work they engaged in for a living posed a risk for the children. The work included farming in the wetlands to grow food such as vegetables and rice. In some cases, even with awareness about risk factors, caregivers had no one to take care of the children when they went to their work places/swamps. When older siblings went to school, the remaining younger ones followed parents to the farms in the swamps or near water bodies.

*Yes, we go with him [to the swamp to work], who else takes care of him! The friends and siblings go to school. I must go with him to the swamp, and he enjoys playing from dirty water when it has rained.* **P7_east central, 30 years**

Caregivers also reported that some of the young children also contributed to the household water needs. Children as young as three years too fetched water in small manageable containers as they were initiated into household chores expected of them later.

*In the process of preparing meals for the family, water is needed, and we send them to fetch it (with older siblings). When they get there, what they do is to first play in the water bodies before bringing water home.* **P18_south western, 59 years**

The caregivers that did not know how schistosomiasis is transmitted also had no hint about how their children could have been infected. Even some that knew how the disease was transmitted could not fathom how their children got infected. These were left shocked at the test results, wondering how this happened.

*How this child got the thing defeated my understanding…I kept on wondering whether the thing (bilharzia) was genetic or something. I thought about food, but she has never taken solid food, she only eats porridge… So how she got this thing (bilharzia) defeated me. If they tell me she could get in my uterus, may be, because some time back I was tested positive with it (bilharzia). Could it have been through blood? I don’t know.* **P25_west Nile, 22 years.**

There were some reported misconceptions about possible ways that children were infected. A few (4/30) caregivers mentioned that their children may have used dirty toilets. Three caregivers thought that the children were probably born with the disease. One caregiver wondered how their child who only bathed river water, acquired the disease.

*My mind was perplexed because I did not know how this child got infected, he does not go to the water body, I do not take him to the water source, I do not bathe him at the water source. Except I fetch the river and use it to bath him at home.* **P30_west Nile, 20 years**

When asked about what stage their children could have been infected, majority (16/30) of the caregivers could not imagine at what stage of growth or possible age this had happened. About one third reported the most probable stage at which the child got infected. The age range suspected was from one year to about three years, although most of the children were 3–4 years old at the time of the study. Caregivers reported that the ages one to three were related to crawling, where children get in contact with dirty surface water (rain water or any stagnant water), mud, possible faecal matter, and playing in swamps and dams during the rainy seasons when some seasonal water bodies have water. Caregivers also argued that at such ages, they provided less care to the children than during infancy. Some said they placed these children under the care of older siblings and did not know whereabouts all the time. There were also cases where caregivers reported that the infection could be congenital.

*“Because during that time (age 2 years) other young children would carry him and go with him to the stream and they also give that water for him to drink”* **P26_west Nile, 19 years.**

*At that stage they play in mud. You bring them in the house and they still find their way out and continue to play in the mud* **P15_south western, 43 years**

### Observation of risky activities exposing children to infection

To triangulate the information from the caregivers’ reports, the research team observed the activities around water bodies in the enumeration areas where the study was conducted. This was intended to establish how the children in the communities potentially get in contact with infested water. The main interest was, therefore, activities that especially included younger children of interest to the study. The research assistants made detailed observations notes from an entire day’s observation for each water body and took photographic evidence. The main activities that were observed included bathing of young children, washing clothes, collecting snail shells for poultry feeds, swimming and playing in the infested water and fetching water. There was also evidence of defecation near water bodies and observed open urination by adults and children.

**Fig. 1:**
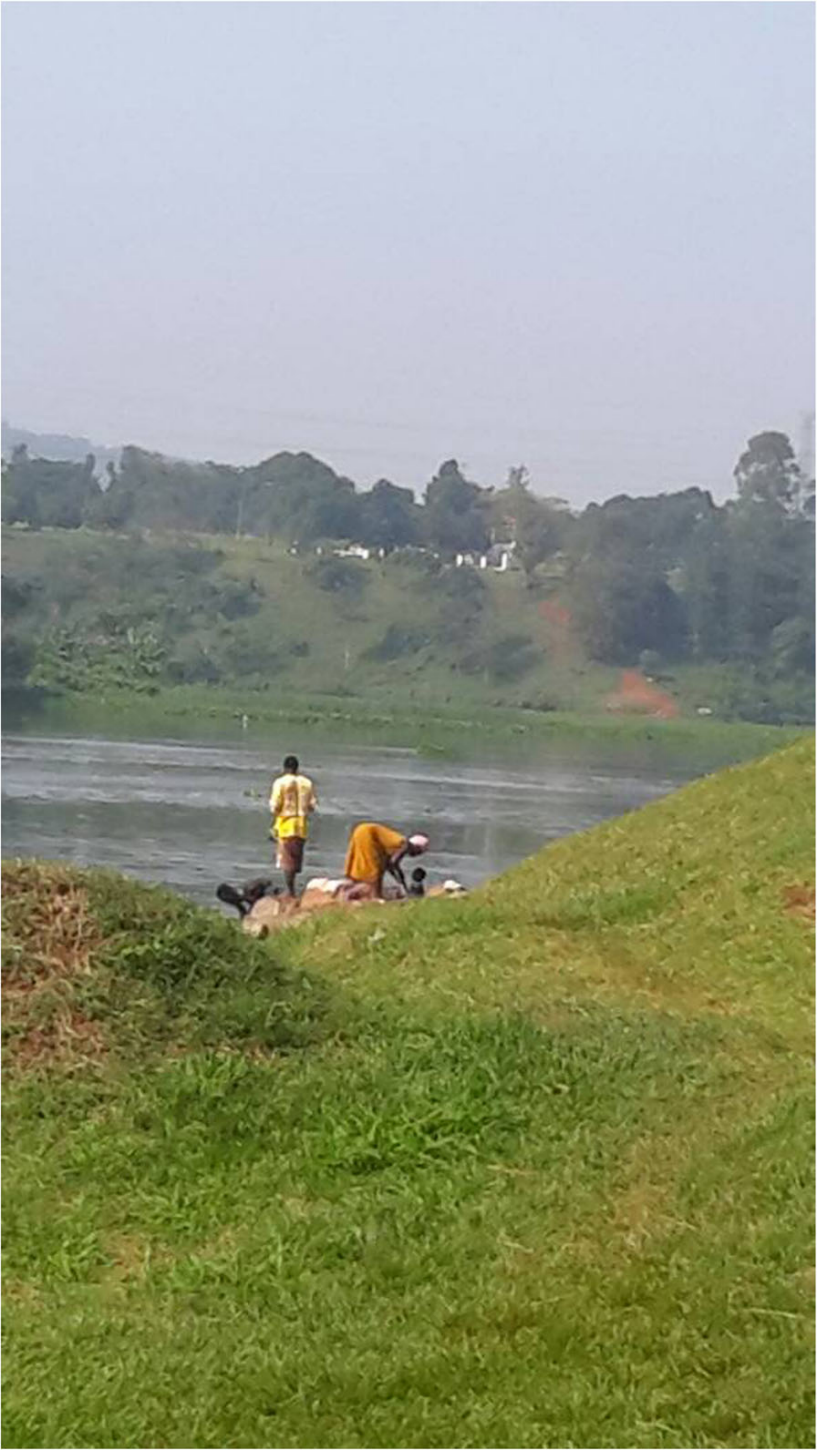
A Child is bathed at the river bank in Jinja, eastern Uganda. A baby is being bathed by the mother using river water collected in a basin. An example of typical water contact activities exposing very young babies that were observed in the villages

Bathing of children was one of the most common activities near the river banks and lake shores in east central and west Nile regions (Fig. 1). The children were either bathed by adults or their older siblings/ children and in some instances on their own while the older children and caregivers watched or were engaged in other activities. In some instances, caregivers were seen bathing their babies at the river banks with water collected from the infested river. The team also observed caregivers cleaning babies in the water bodies after they had defecated nearby.

Washing clothes was a common sight at both riverbanks and lake shores. In west Nile, while caregivers washed clothes during the scotching sun, crying babies were soothed by sitting them in a basin of the water collected from the river. This enabled the caregiver to continue washing clothes with less distraction, while the baby played in the basin. The younger children also played in the shallow waters while their older siblings and caregivers washed clothes. Some were seen climbing into non-functional boats abandoned at the lake shores in east central Uganda. They collected stagnant water from these boats and played water games, pouring it on to their colleagues and babies. These younger ones played in the water under the watch of the older teenage siblings/ colleagues.

Swimming was a popular recreation activity that was observed from the older children in the company of their younger siblings. They were seen swimming especially from their “gazetted” areas. In west Nile (Arua), swimming was very popular because of the scotching sun. The children generally went to swim in large groups. Boys and girls of ages ranging from about three to 12 years situated themselves at different sides of the river; many undressed-on arrival and went into the river right away.

There was open urination near or in the water bodies. Men and boys were more commonly observed urinating by the water body in some areas prior to diving into the water to bathe or swim. This was the same water that the other children played in and babies were bathed. There was also evidence of open defecation around the water bodies and catchment areas, as well as children going into the nearby bushes to relieve themselves. This could be because they played in the water for extended periods, yet there were no latrines around the water bodies to ease themselves. Human faeces were observed floating in one of the rivers. Decomposing faeces were also seen in farmland around water bodies.

Adults and older children fetched water for household use from the shallow end of the rivers and lake. It was thus easy for them to step directly into the river to fetch water. They used the basins to collect water for washing clothes; saucepans for washing utensils; and jerrycans to collect water for domestic use. In their company were usually the younger children; about four and 8 years. Fetching water seemed to be a communal activity in all the areas that were observed in West Nile and East central regions. Women moved to the water bodies in groups, with their children. The older children had the responsibility of taking care of the younger ones; keeping watch to ensure their safety by the water.

## Discussion

This qualitative study conducted among caregivers of children positive for schistosomiasis in the East-central, West Nile and South western sub-regions of Uganda, explains how schistosomiasis infection in endemic areas in Uganda begins at a very early age. The exposures from caregivers’ accounts triangulated with direct observations suggest that many children under 5 years in endemic areas are frequently in contact with infested water. Yet, this age group is not a target for mass drug administration. This could explain why the children 2–4 years had the highest prevalence of schistosomiasis in the National surveys in Uganda. It also shows challenging gaps in knowledge about how schistosomiasis is transmitted with caregivers of children in this study concurrently having the right information, but also varied misconceptions.

Caregivers in this study had mixed information about schistosomiasis and how it is spread. Only a few caregivers did not have any knowledge about schistosomiasis transmission. It is encouraging that most caregivers had knowledge about potential sources of infection. Such knowledge needs to be enhanced, well knowing that it filters to other community members through word of mouth. Amidst the right information were also misconceptions that need to be allayed. The existence of the right information without tackling wrong beliefs may continue to facilitate schistosomiasis infection or negate treatment efforts. Misconceptions about transmission were rife including thoughts that it was through witchcraft, and that directly bathing children with river water fetched in containers was not a risk factor. Some misconceptions and gaps in knowledge have been quantified in Uganda [15, 21] and elsewhere [22]. This is glaring evidence that the caregivers and probably many people in the endemic communities are not comprehensively knowledgeable about the modes of transmission for the schistosomiasis. Sources of information such as the radio, health workers and schools and village health teams were commonly mentioned. These are some of the avenues that the Ministry of Health’s Vector Control Division and Schistosomiasis control partners use to convey the right messages. Indeed, some of the messages mentioned by caregivers in this study were those relayed in a recent campaign before the 2017 survey. However other less reliable sources like community members were also mentioned, and these may have altered messages.

The observed risky activities including open defecation and urination near and in water bodies show a glaring lack of proper sanitation facilities in several communities. Many households in study communities were living in poverty and may need support for safe water and sanitation facilities. This will primarily avoid contact with schistosome infested water and secondly reduce contamination of the water bodies. Environmental exposures such as contaminated water are not only a risk factor for schistosomiasis but also affect the health of children in many other ways [23]. Improvements in water and sanitation will, therefore, impact on the overall health and welfare of children and contribute to the achievement of the Sustainable Development Goals (SDGs) especially goal 3 [24] as has been suggested before [21].

Some of the activities that exposed children to the risk of infection are vital to the survival of the caregivers and ultimately the children they are trying to protect. This means that messages that only aim at preventing contact with infested water may not have an effect in such cases. Some caregivers, for example, went with their children to their work-places such as swamps where they practised farming. This was because they had no one to care for the children while they went to win bread for their families. In other cases, younger children had to contribute to household water needs together with their siblings, which is vital to the survival of households. This further supports the evidence for the need for universal coverage of sanitation facilities to prevent communities from infecting water bodies. There is clear evidence of a lower prevalence of schistosomiasis in areas where sanitation facilities are most used (16%) versus those where open defecation and urination are rife (28%) [15].

In order to contribute to the global target to control schistosomiasis by the year 2020 [1], there is need to ensure treatment access to all ages [17, 25, 26]. If nothing is done among children under 5 years like those presented in this study, the efforts to control and eliminate schistosomiasis will definitely be hampered [6]. For example, children under age five comprise 18% of Uganda’s population [27] and can thus be a big “reservoir” of infection. Even when mass treatment of adults and school-age children is done, these children under 5 years will continue to be a big source of transmission in endemic areas, given their contact with water bodies. This is also likely to apply to other endemic countries whose fertility rates are still high and a big proportion of the population are children. In fact, Mutapi et al. reported that children age 1 to 5 years in Zimbabwe carry infection levels that are higher than those of their guardians/parents [17].

As developments into child size medicines for use in mass drug administration and vaccines [8, 28, 29] continue, the most effective interventions must emphasise prevention of infection. Targeted specific messages for caregivers of children need to be developed and spread nationwide to prevent infection among young children, who are not eligible for mass drug administration. Such messages may also need to be context specific. For example, erecting signages near infested water points accessed by communities, to warn caregivers from exposing their children may be helpful. Other messages are already running in endemic areas in Uganda about non-contact with water and boiling water before using it at home. However, these need to be expanded to the entire country and tailor-made for adults, children who can listen and understand, as well as caregivers for their children. Emphasis in these health promotion messages about challenges in child medicines [12] or non-availability of mass treatment of children below 5 years in Uganda may help prompt caregivers to avoid exposure of their children to potentially infested waters and improve hygiene. The worry expressed in this study among caregivers whose children could not receive treatment is an indication that such messages may influence behaviour change.

### Limitations

The study findings should be interpreted in the context of some limitations. The in-depth interviews are based on caregivers recall about activities that their children engaged in that could have exposed them to schistosomiasis. There is a possibility of recall bias. However, interviewing them after a positive test result was confirmed, could have improved their recall about activities that they would otherwise not have considered risky before. The study also used observations to triangulate the evidence from the accounts of the caregivers. The observed activities conformed with caregivers’ reports.

## Conclusions

This study shows that many pre-school children in schistosomiasis endemic areas of Uganda are constantly exposed to Schistosoma infested water during activities that involve their adult caregivers, older siblings in the households and friends in their communities. It provides in-depth explanation about how children get infected at an early age and provides more evidence for the need to include pre-school children in prevention and treatment efforts.

## Abbreviations

NTDs: Neglected Tropical Diseases
POC-CCA: Point of care circulating cathodic antigen
SDGs: Sustainable Development Goals
WHO: World Health Organisation
